# Surgomics: personalized prediction of morbidity, mortality and long-term outcome in surgery using machine learning on multimodal data

**DOI:** 10.1007/s00464-022-09611-1

**Published:** 2022-09-28

**Authors:** Martin Wagner, Johanna M. Brandenburg, Sebastian Bodenstedt, André Schulze, Alexander C. Jenke, Antonia Stern, Marie T. J. Daum, Lars Mündermann, Fiona R. Kolbinger, Nithya Bhasker, Gerd Schneider, Grit Krause-Jüttler, Hisham Alwanni, Fleur Fritz-Kebede, Oliver Burgert, Dirk Wilhelm, Johannes Fallert, Felix Nickel, Lena Maier-Hein, Martin Dugas, Marius Distler, Jürgen Weitz, Beat-Peter Müller-Stich, Stefanie Speidel

**Affiliations:** 1 Department of General, Visceral and Transplantation Surgery, Heidelberg University Hospital, Im Neuenheimer Feld 420, 69120 Heidelberg, Germany; 2 National Center for Tumor Diseases (NCT), Heidelberg, Germany; 3 Department of Translational Surgical Oncology, National Center for Tumor Diseases (NCT/UCC), Dresden, Germany; 4 Cluster of Excellence “Centre for Tactile Internet with Human-in-the-Loop” (CeTI), Technische Universität Dresden, 01062 Dresden, Germany; 5 Corporate Research and Technology, Karl Storz SE & Co KG, Tuttlingen, Germany; 6 Department of Visceral-, Thoracic and Vascular Surgery, University Hospital Carl Gustav Carus, Technische Universität Dresden, Dresden, Germany; 7 Institute of Medical Informatics, Heidelberg University Hospital, Heidelberg, Germany; 8 Research Group Computer Assisted Medicine (CaMed), Reutlingen University, Reutlingen, Germany; 9 Department of Surgery, Faculty of Medicine, Klinikum Rechts der Isar, Technical University of Munich, Munich, Germany; 10 Department of Intelligent Medical Systems (IMSY), German Cancer Research Center (DKFZ), Heidelberg, Germany; 11 National Center for Tumor Diseases (NCT/UCC), Dresden, Germany; 12 German Cancer Research Center (DKFZ), Heidelberg, Germany; 13 Faculty of Medicine and University Hospital Carl Gustav Carus, Technische Universität Dresden, Dresden, Germany; 14 Helmholtz-Zentrum Dresden - Rossendorf (HZDR), Dresden, Germany; 15 Else Kröner Fresenius Center for Digital Health, Technische Universität Dresden, Dresden, Germany

**Keywords:** Artificial intelligence, Minimally invasive surgery, Radiomics, Prediction model, Surgical data science, Precision medicine

## Abstract

**Background:**

Personalized medicine requires the integration and analysis of vast amounts of patient data to realize individualized care. With Surgomics, we aim to facilitate personalized therapy recommendations in surgery by integration of intraoperative surgical data and their analysis with machine learning methods to leverage the potential of this data in analogy to Radiomics and Genomics.

**Methods:**

We defined Surgomics as the entirety of surgomic features that are process characteristics of a surgical procedure automatically derived from multimodal intraoperative data to quantify processes in the operating room. In a multidisciplinary team we discussed potential data sources like endoscopic videos, vital sign monitoring, medical devices and instruments and respective surgomic features. Subsequently, an online questionnaire was sent to experts from surgery and (computer) science at multiple centers for rating the features’ clinical relevance and technical feasibility.

**Results:**

In total, 52 surgomic features were identified and assigned to eight feature categories. Based on the expert survey (*n* = 66 participants) the feature category with the highest clinical relevance as rated by surgeons was “surgical skill and quality of performance” for morbidity and mortality (9.0 ± 1.3 on a numerical rating scale from 1 to 10) as well as for long-term (oncological) outcome (8.2 ± 1.8). The feature category with the highest feasibility to be automatically extracted as rated by (computer) scientists was “Instrument” (8.5 ± 1.7). Among the surgomic features ranked as most relevant in their respective category were “intraoperative adverse events”, “action performed with instruments”, “vital sign monitoring”, and “difficulty of surgery”.

**Conclusion:**

Surgomics is a promising concept for the analysis of intraoperative data. Surgomics may be used together with preoperative features from clinical data and Radiomics to predict postoperative morbidity, mortality and long-term outcome, as well as to provide tailored feedback for surgeons.

**Graphical abstract:**

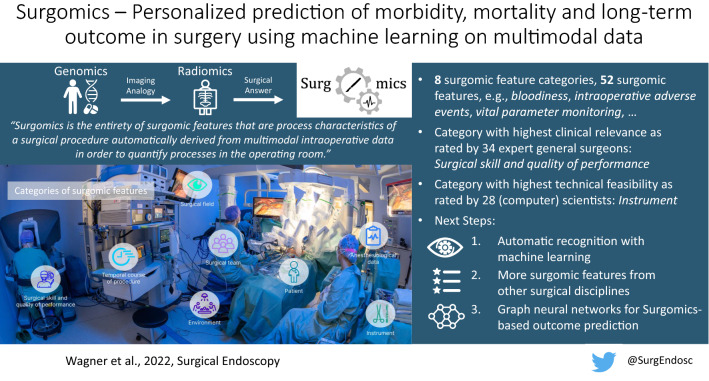

**Supplementary Information:**

The online version contains supplementary material available at 10.1007/s00464-022-09611-1.

Surgery is an important building block of the treatment for a multitude of diseases, for example in multivessel coronary artery disease [1] or solid cancer [2]. At the same time, surgery is dangerous with postoperative death being the third leading cause of death worldwide [3]. The complexity of surgical treatment results from a multitude of interactions between patients, clinical staff members and medical devices during pre-, intra- and postoperative phases. Optimal surgical results that can theoretically be achieved are not always reached in daily practice. Especially during the postoperative treatment pathway, complications like erosive bleeding from a pancreatic fistula [4] or mediastinitis after an esophageal anastomotic leakage [5] can occur. These complications result in a significant deterioration of the patient’s outcome or even in the death of the patient. Patient-associated factors such as age, sex, body mass index (BMI) or comorbidities such as cardiac disease or diabetes can have a large impact on the postoperative outcome and may therefore be used for outcome prediction [6, 7]. But also surgeon and hospital experience [8], as well as an adequate treatment of major postoperative surgical complications are influencing the mortality rates in different perioperative care systems [9]. With regard to the intraoperative setting, increasing evidence suggests that specific parameters and events are strongly associated with postoperative complications and outcome [10]. For example, intraoperative factors like a staple line bleeding in laparoscopic distal pancreatectomy can be a potential precursor of a later pancreatic fistula, as demonstrated using video review-based analysis [11]. Furthermore, it has been proven that surgical skill and technical performance are strongly associated with postoperative clinical outcome [12–14]. Nevertheless, the predictive accuracy of surgeons for postoperative complications appears limited, e.g. in case of anastomotic leakage in gastrointestinal surgery [15].

In non-surgical areas of multimodal treatment especially for cancer, personalized therapy recommendations along the treatment pathway have already been pursued with the approaches of Genomics, Epigenomics, Transcriptomics, and Microbiomics in (tumor) biology [16], and with Radiomics in clinical medicine [17]. However, an omics-approach in surgery that uses intraoperative data comprehensively, i.e. combines surgical data specific to the operating room to create a quantitative description of a surgical procedure is not yet established. This deficiency may result from the large scale and velocity of multimodal data in the operating room produced by heterogeneous sources of raw data, or might also be related to the high level of variability of surgical processes [18]. To pave the way for a personalized therapy, which makes use of intraoperative data, relevant surgical process characteristics need to be extracted from data sources like endoscopic videos, intraoperative imaging methods or vital sign monitoring using machine learning (ML) methods [18]. First steps in this endeavor have been made in the preoperative setting using ML to automatically identify high risk surgical patients [19]. Intraoperatively, ML has been widely studied for the recognition of surgical phases [20, 21], for the automatic assessment of the surgeon’s skill level [22], or for predicting postoperative adverse events and distractions [23]. With the OR black box, Jung et al. presented a first comprehensive approach of quantitatively identifying intraoperative events and distractions by collecting intraoperative data [24]. Based on this work, they developed an index to measure the severity of intraoperative events and thus identified patients at high risk of developing postoperative complications [25]. However, their analysis was based on human expert ratings and not on a computer-based approach, thus requiring a huge effort and time limiting its scalability. Up until now ML has not been applied within a comprehensive and concrete approach to use intraoperative surgical data to support surgical decision-making leading to a lack of success stories in the field of surgical data science [26]. We argue that the absence of a useful and classified representation of recorded intraoperative data, which would allow for the analysis of vast amounts of heterogeneous data is one major reason in this regard.

The aim of this article is to approach this shortcoming by defining the concept of Surgomics as a surgical answer to Genomics and Radiomics. After identifying and categorizing intraoperative surgomic features, this article investigates which surgomic features are perceived as clinically most relevant and technically most feasible by surgeons and (computer) scientists with a focus on the example of minimally invasive oncological surgery. In contrast to the general concept of surgical data science, Surgomics will focus on the intraoperative setting to use and integrate the rapidly increasing amounts of intraoperative sensor data. Surgomics may be used together with pre- and postoperative data to improve surgical treatment with surgical data science methods. Furthermore, Surgomics could enable digital surgery applications such as decision support systems, computer-assisted training, and robotics [27].

## Materials and methods

We started with the idea to systematically explore intraoperative data and use methods of feature extraction from those vast amounts of data similar to Radiomics. In order to elaborate this raw idea we brought together experts from multiple surgical and scientific disciplines and came up with a definition “Surgomics”, i.e. its data sources, methods and applications. Then, we discussed ideas for surgomic features, categorized them, and used what we found to eliminate uncertainties in the definition of Surgomics. Finally, we validated the ideas for features and their relevance in a multicenter expert survey.

### Definition of Surgomics

We define Surgomics as follows: *Surgomics is the entirety of surgomic features that are process characteristics of a surgical procedure automatically derived from multimodal intraoperative data to quantify processes in the operating room. Surgomics is thus the surgical answer to Genomics and Radiomics and may be used together with preoperative features from clinical data science and Radiomics to predict postoperative morbidity, mortality and long-term outcome as well as to provide tailored feedback for surgeons.*

**Fig. 1:**
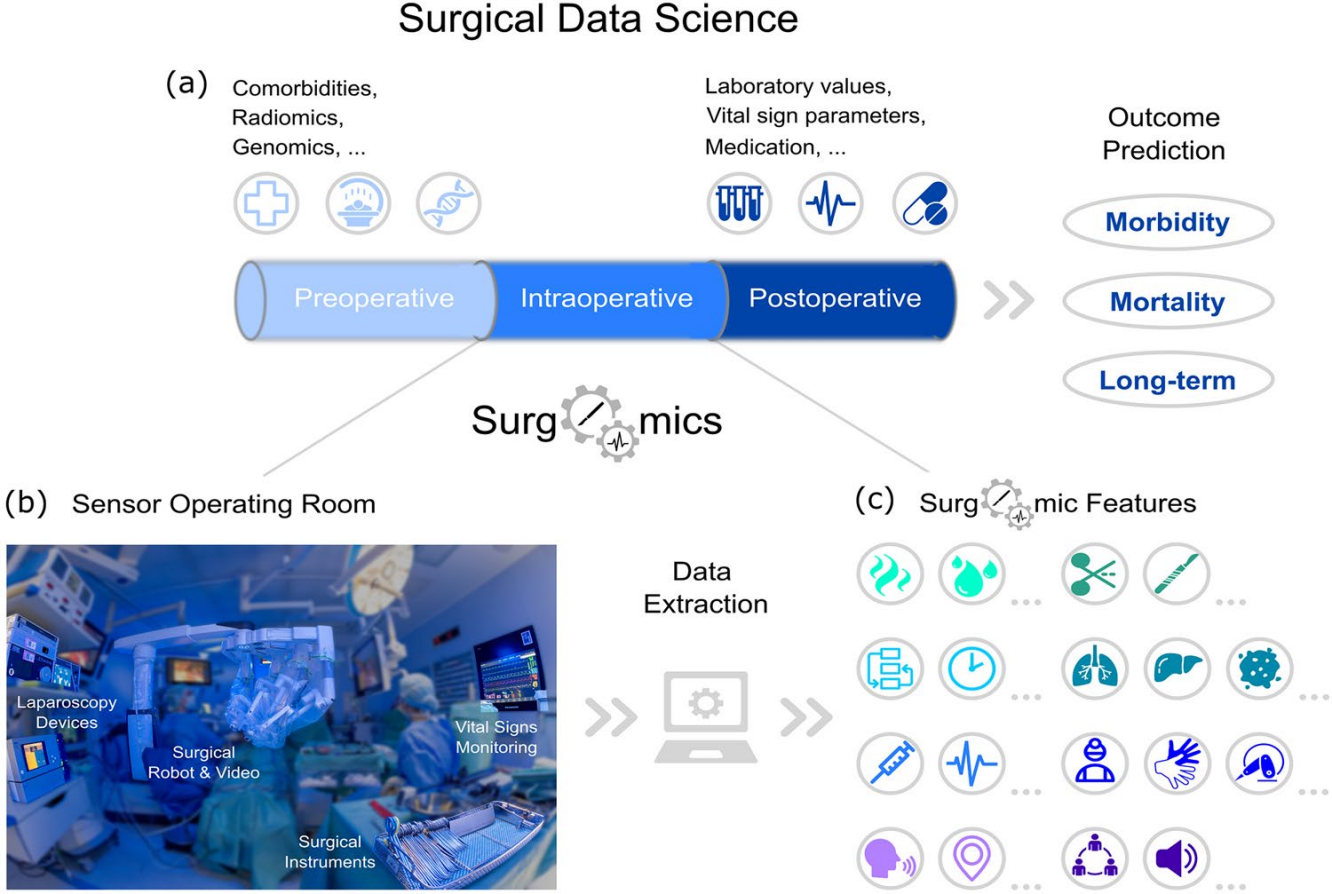
Concept of Surgomics. **a** In surgical data science, pre-, intra- and postoperative data are integrated to predict morbidity, mortality and long-term outcome. **b** Surgomics focuses on the intraoperative setting that comprises data sources like the surgical video or anesthesiological vital sign monitoring. **c** Surgomic features can be extracted from suitable data sources in an automated fashion, for example using machine learning or other data science methods

Based on previous work in surgical data science [18], surgical process models [28], surgical workflow analysis [29], and health information networks [30], we aim to establish Surgomics as a concept to collect, process and structure multimodal data in the intraoperative setting (Fig. 1). To reach this goal, surgical expert knowledge needs to be combined with ML and data science methods to make effective and scalable use of the vast amounts of intraoperative data.

### Categorization of surgomic features

**Fig. 2:**
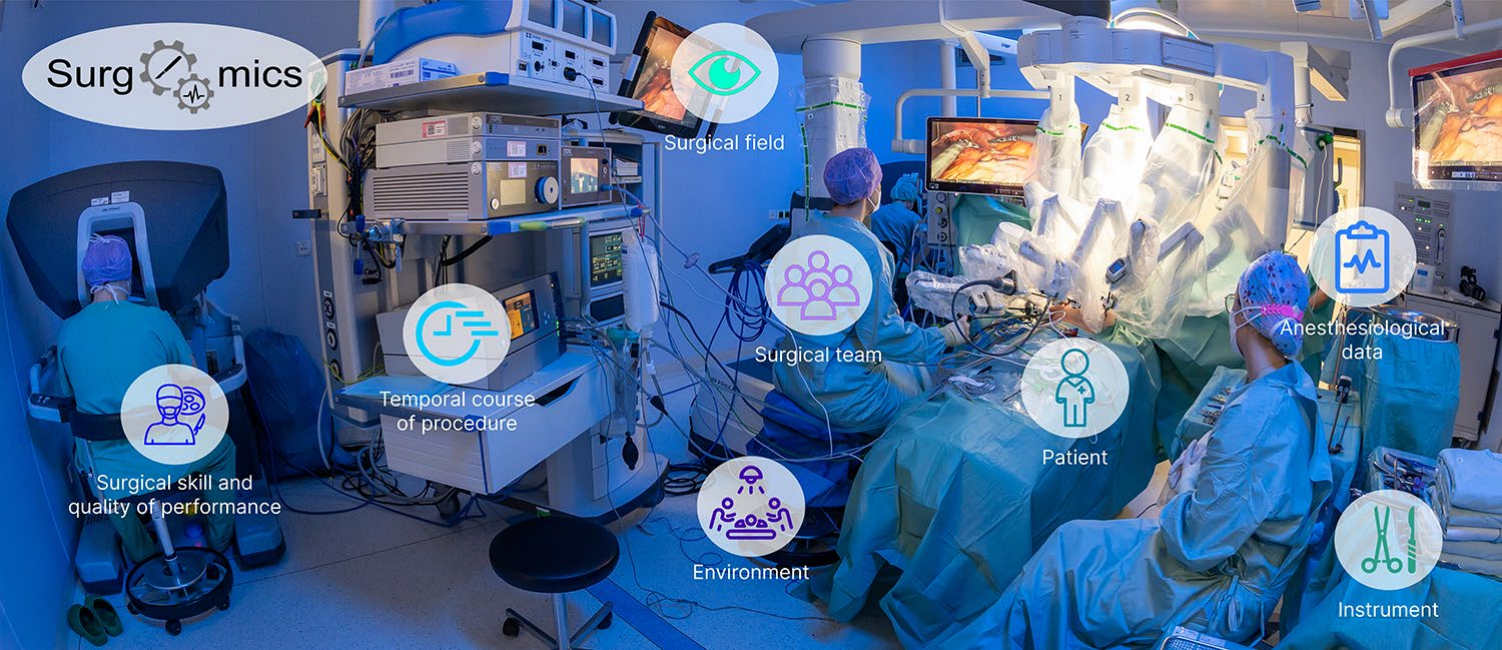
Categories of surgomic features. A team of multidisciplinary experts defined eight feature categories to classify surgomic features

The aforementioned definition of Surgomics and surgomic features resulted as a first step in development by a multidisciplinary team (*n* = 19) consisting of experts from the fields of colorectal, pancreatic, upper gastrointestinal, robot-assisted and minimally invasive surgery on one hand, as well as translational surgical oncology, surgical data science, connected medical devices, medical informatics, medical data models, clinical decision support, radiomics, and endoscopic vision on the other hand. In a next step, the multidisciplinary team discussed the variety of data available in the intraoperative setting and their potential relevance for the patient's short-term surgical outcome (morbidity and mortality), as well as long-term outcome (survival). In the course of the discussion, examples of surgomic features were collected and categorized. Here, we aimed for a broad spectrum of features using different intraoperative data sources to foster future discussion on new features. Later on, additional information for each feature was added, i.e. unit of measurement, source of raw data, specificity for a certain surgical procedure and literature references if applicable (Table 1). Regarding the categorization of the surgomic features, the multidisciplinary team agreed on the following eight feature categories (Fig. 2):

1. Surgical field
  The surgical field category contains surgomic features which can be derived from the surgical video (for example in laparoscopy or thoracoscopy) and directly apply to the surgical procedure, for example the level of blood and smoke in the surgical field or imaging methods like the accumulation of indocyanine green (ICG) as a measure for the perfusion in an anastomosis.
2. Instrument
  The instrument category comprises features related to surgical instruments, e.g. the instrument location in the surgical video, the action performed with an instrument or medical device, device settings such as voltage in electrosurgery, or vacuum and pressure settings for suction and irrigation. The source of raw data can be the (endoscopic) video, but data can also be derived directly from the medical device sensor connected to an instrument.
3. Temporal course of procedure
  The category relating to the temporal course of procedure contains characteristics, which describe the surgery with respect to its workflow, e.g. the duration and order of performed surgical steps.
4. Patient
  The patient category represents patient-specific characteristics such as the visual texture of lung or liver derived from surgical video, or the pancreatic duct diameter. These features are derived directly from the patient and may also be available preoperatively, e.g. from computed tomography.
5. Anesthesiological data
  Examples for features in the anesthesiological data category are data of the blood gas analysis or the vital sign monitoring. The features can be derived from the (electronic or digitized) anesthesia protocol or from anesthesiological devices.
6. Surgical skill and quality of performance
  The category referring to the surgical skill and quality of performance contains features, which are specific to each surgeon. For instance, surgical skill can be objectified with the GOALS score [31] including features such as bimanual dexterity or tissue handling. Other features occurring in the course of surgery, directly depending on the operating surgeon, are the quality of a suture or the extent of dissection of lymphatic tissue in oncological surgery [32].
7. Surgical team
  These features depend on the constellation and interaction of the surgical team like the position of the different team members around the patient or the quality of team communication. Additionally, characteristics, which are individual for each team member, like their experience in the respective surgical procedure or their current stress level as measured by cortisol level or heart rate, are included in this category. Furthermore, team aspects such as the number of procedures that two or more members of the surgical and/or anaesthesiological team have performed together prior to the operation, can be considered within this category. However, data sources to collect intraoperative information on the team like a microphone or a room camera in the OR have to be used with caution considering data privacy concerns.
8. Environment
  Features regarding the environmental setting of the OR containing metadata of the surgery are summarized in this category. Examples are the emotional atmosphere, the noises, or the temperature in the operating room. Again, a microphone or a room camera could be used for data acquisition.

**Table 1:** Surgomic features

No	Feature	Description and use case	Feature category	Source of raw data	Procedure specific?	Relevant reference
1	Bloodiness	How bloody is the surgical field based on a predefined scale, e.g., five options, one of them “small amount of blood”. Based on this feature, other information like the number of bleedings requiring hemostasis can be calculated	Surgical field	Video	No	[36]
2	Smokiness	How smoky is the surgical field based on a predefined scale, e.g., four options one of them “no visibility due to the amount of smoke”	Surgical field	Video	No	[37]
3	Presence of anatomic structure	E.g., is the gastric tube or the azygos vein visible in the video?	Surgical field	Video	No	[38]
4	Location of anatomic structure	Where is an anatomic structure like the gastric tube located? What is the 3D position of the anatomic structure?	Surgical field	Video	No	[39]
5	Action performed with anatomic structure	Which action is performed with the anatomic structure and how often or for how long has the action occurred? E.g., the gallbladder has been grasped ten times	Surgical field	Video	No	[40, 41]
6	Colors of anatomic structures	E.g., does the color of the gastric tube look pale or vital?	Surgical field	Video	No	
7	Indocyanine green accumulation curve of perfusion in anatomic structures	How well is the esophagogastrostomy perfused based on the ICG-accumulation?	Surgical field	Video	Yes	[42]
8	Hyperspectral imaging of anatomic structures	How well is the perfusion of an organ as measured by hyperspectral imaging ?	Surgical field	Hyperspectral imaging device	Yes	[43]
9	Intraoperative adverse events	Which intraoperative adverse events like a major vessel injury or a re-do anastomosis do occur during the procedure?	Surgical field	Video, anesthesia protocol, surgery report	No	[23, 25, 44]
10	Presence of instrument	E.g., is the suction, clip-applier or vessel sealer visible in the video?	Instrument	Video	No	[46]
11	Location of instrument	Where is an instrument like the grasper located? What is the 3D position of the instrument?	Instrument	Video, robot	No	[22]
12	Action performed with instrument	Which action is performed with the instrument? How often or for how long has the action occurred? E.g., the vessel sealer is used for 30 min	Instrument	Video	No	[41, 47]
13	Instrument usage	Which pattern does the instrument usage show? E.g., for how long and in which sequence has the instrument been used? How many tool changes occur or in which combination with other instruments is the tool used?	Instrument	Video, device data	No	[48]
14	Instrument open/closed	Is the instrument, e.g., the scissors, open or closed?	Instrument	Video, robot	No	
15	Number of camera cleanings	How often has the camera been cleaned during the surgery?	Instrument	Video	No	
16	Instrument pressure or flow	How strong is e.g., the insufflator flow over time?	Instrument	Device data	No	[49]
17	Duration of surgery	How long does the surgery take? How long do the different surgical phases and steps, e.g. the gastric tube construction take?	Temporal course of procedure	Video	No	[50]
18	Order of performed phases/steps	In which order are the surgical phases or steps performed? Are there deviations from the normal order?	Temporal course of procedure	Video	No	[20, 50]
19	Duration of inside/outside sequences	When is the camera outside of the patient’s body? How long do these outside sequences take?	Temporal course of procedure	Video	No	
20	Texture of lungs	How does the texture of the lung appear, e.g., what is the smoker status of the patient?	Patient	Video	No	[51]
21	Texture of liver	E.g., does the patient’s liver show cirrhosis or metastases? How does the liver texture appear e.g., after a chemotherapy?	Patient	Video	No	[52]
22	Texture of pancreatic gland	How does the texture of the pancreatic gland appear, e.g. are there signs of atrophy or chronic inflammation?	Patient	Video	No	[53, 54]
23	Frozen section result	Is the frozen section result tumor free or are still malignant cells present?	Patient	Pathology protocol	Yes	[55]
24	Gastric tube diameter	How wide is the diameter of the gastric tube estimated intraoperatively during an esophagectomy?	Patient	Video	Yes	
25	Pancreatic duct diameter	How wide is the diameter of the pancreatic duct estimated intraoperatively?	Patient	Video	Yes	[54]
26	Volume of remnant gland	How large is the volume of the pancreatic gland at the end of a pancreatic surgery?	Patient	Video	Yes	
27	Drug administration	Which medication is administered to the patient during surgery?	Anesthesiological data	Anesthesia protocol	No	
28	Blood gas analysis	Does the patient e.g. develop a metabolic acidosis during surgery?	Anesthesiological data	Anesthesia protocol	No	
29	Vital sign monitoring data	How are the vital signs of the patient during the surgery, e.g. the arterial blood pressure curve?	Anesthesiological data	Anesthesia protocol, device	No	[56]
30	Difficulty of surgery	How difficult is the surgery compared to other surgeries of the same type, e.g., are there aberrant vessels present or are there a lot of abdominal adhesions?	Surgical skill and quality of performance	Video, sound recording, …	No	[57]
31	Depth perception	How well does the surgeon operate with the laparoscopic or robotic optical system, e.g., are there frequent overshootings of the instruments?	Surgical skill and quality of performance	Video	No	GOALS-Score [31]
32	Bimanual dexterity	How well does the surgeon use both hands, e.g., does he or she frequently ignore the nondominant hand?	Surgical skill and quality of performance	Video	No	GOALS-Score [31]
33	Efficiency	How fluent is the surgeon in his or her actions, e.g. does the surgeon often switch from one area of dissection to another?	Surgical skill and quality of performance	Video	No	GOALS-Score [12, 31]
34	Tissue handling	Does the surgeon handle tissue gently and is the use of laparoscopic or robotic instruments appropriate, e.g., does he or she adapt to the loss of tactile feedback?	Surgical skill and quality of performance	Video	No	GOALS-Score [12, 22, 31]
35	Autonomy	How much guidance of a more experienced surgeon does the operating surgeon need, e.g. does the more experienced surgeon needs to take over the operation?	Surgical skill and quality of performance	Video, sound recording	No	GOALS-Score [31]
36	Tissue exposure	How well is the tissue exposed for the performed surgical action, e.g. the critical view of safety in cholecystectomy?	Surgical skill and quality of performance	Video	No	[12]
37	Suture quality of anastomosis	E.g., is the approximation of the sutures appropriate?	Surgical skill and quality of performance	Video	Yes	[32]
38	Extend of dissection/resection	How well have anatomic structures like the aorta been dissected? How large is the extent of removed lymphatic tissue?	Surgical skill and quality of performance	Video	No	[32]
39	Tension of anastomosis	Is there tension at an anastomosis, e.g., an esophagogastrostomy, visible?	Surgical skill and quality of performance	Device	Yes	[32]
40	Force of stomach pull-up	E.g., was excessive force used during the stomach-pull up in an esophagectomy?	Surgical skill and quality of performance	Device	Yes	
41	Number of team members	How many people are in the operating room and who are part of the surgical team? How does the number of team members change over time? Are more people present during specific surgical steps than during others?	Surgical team	OR camera	No	[24]
42	Position of team members	Where are the different surgical team members located in the operating room? How does the constellation change during surgery?	Surgical team	OR camera	No	[58]
43	Quality of team communication	How do different surgical team members interact and communicate with each other, e.g., are there often misunderstandings or in which moments do distractions occur?	Surgical team	Sound recording	No	[24]
44	Fastness of decision-making	If an intraoperative adverse event occurs, how and how fast does the surgical team make a decision?	Surgical team	Sound recording	No	[59]
45	Surgeries performed as a team	How many surgeries has the surgical team performed in this constellation?	Surgical team	Past OR schedules	No	
46	Familiar surgeries performed / Experience with surgery	How many robotic or laparoscopic surgeries has a surgical team member already performed? What is the overall experience of the team member with robotic or laparoscopic surgery? How many surgeries of exactly the present surgery, e.g. robot-assisted esophagectomies has a surgical team member already performed?	Surgical team	Past OR schedules	No	[60]
47	Stress level	How high is the stress level of the surgical team members? This could be measured by the cortisol level	Surgical team	Blood sample	No	
48	Heart rate	How high is the heart rate of the surgical team members?	Surgical team	Device	No	
49	Voice frequency	How high is the voice frequency of the surgical team members?	Surgical team	Device	No	[59]
50	Emotional atmosphere in OR	How is the emotional atmosphere in the operating room? E.g., could the atmosphere be tense after an adverse event occurred connected with more noises and a rather harsh language	Environment	Sound recording	No	[24]
51	Temperature in OR	How high is the temperature in the operating room?	Environment	Device	No	
52	Noises in the OR	Which noises do occur during the procedure and how loud are those noises?	Environment	Sound recording	No	[59]

List of possible surgomic features with description, use case, and category for each feature. Additionally, the source of raw data and the specificity for a certain surgical procedure is shown for each feature. If applicable, a reference is added describing a possible method to assess and/or use the feature, or closely related work

**Fig. 3:**
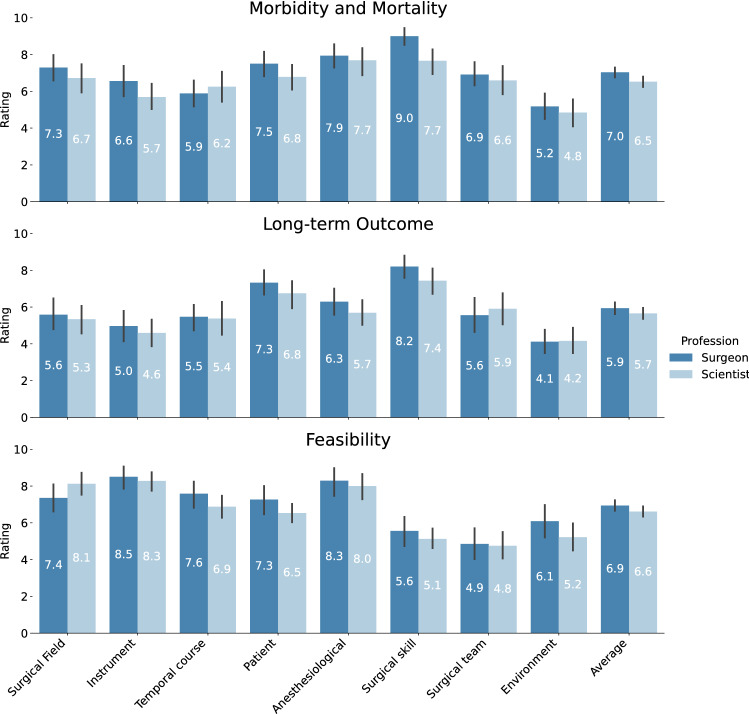
Rating of surgomic feature categories. Ratings are displayed in each subplot per feature category for clinical relevance regarding morbidity and mortality, clinical relevance regarding long-term (oncological) outcome and technical feasibility. Colors depict ratings of surgeons and scientists, respectively. The only significant difference between surgeons and scientists was in the category “surgical skill and quality of performance” regarding the relevance for morbidity and mortality (*p* = 0.002)

### Multicenter expert survey

In the next step a questionnaire presenting all surgomic feature categories and their respective features was developed to validate and rate them. The questionnaire was implemented and analyzed with the online tool LimeSurvey (LimeSurvey GmbH, Hamburg, Germany) hosted by Heidelberg University (Heidelberg, Germany). We sent out the questionnaire to the departments of general and visceral surgery at three German university hospitals, six German research groups from computer science, the section for computer and telematic assisted surgery of the German Society of Surgery (DGCH), and participants of the endovis-challenges HeiChole 2019 and HeiSurf 2021 (https://endovis.grand-challenge.org/).

The opening page of the questionnaire explained the concept of Surgomics and mentioned the different partners involved in the project.

The participants were then asked for their gender, professional background (general or visceral surgeon, other surgical specialty, computer scientist, scientist from other discipline, other) and years of professional work experience since graduation (for surgeons since beginning of residency).

In the next step the participants were asked to rate the eight feature categories on a numeric rating scale from 1 to 10 (1 = not relevant/feasible; 10 = extremely relevant/feasible) regarding their:

1. Clinical relevance for morbidity ("patient suffers postoperative complications") and mortality ("patient dies within 90 days after surgery"),
2. Clinical relevance for the long-term oncological outcome ("survival, e.g. postoperative survival 10 years after surgery"),
3. Technical feasibility of automatic recognition with machine learning ("Artificial Intelligence").

Each feature category was displayed with at least one example. If the participant needed more information about a category, there was the option to hover over the category and get a more detailed description displayed.

Finally, participants were asked to rank the 52 available surgomic features within their respective category (Table 1) via drag and drop regarding their overall priority. Again, if the participant needed more information about a specific surgomic feature, there was the option to hover over the feature and display a more detailed description. Additionally, participants were invited to add missing surgomic features in a free text field.

### Statistical analysis

The results of the survey were analyzed using descriptive statistics with Python 3.10 using pandas 1.4, scipy 1.8 and seaborn 0.11, (Python Software Foundation [33]). For the surgomic feature categories mean and standard deviation were calculated for clinical relevance and technical feasibility. For the priority ranking of the surgomic features in each category mean ± standard deviation of the rank were calculated. Results were calculated separately for surgeons and (computer) scientists. A test for normal distribution was performed with the Shapiro–Wilk-test [34] and results were compared with Welch's unequal variances *t* test [35].

## Results

Overall, 52 intraoperative surgomic features have been identified in the multidisciplinary panel discussion. Table 1 gives an overview of the surgomic features and their properties. Most features were assigned to the “surgical skill and quality of performance” category (*n* = 11). The categories “temporal course of procedure”, “anesthesiological data” and “environment” had the least features (*n* = 3 each). The categories “surgical field” and the “surgical team” (*n* = 9 each) as well as “instrument” and “patient” (*n* = 7 each) had intermediate numbers of features. Overall, *n* = 9 (17%) features were procedure specific, meaning that they cannot be derived and analyzed in every surgery like the ICG-accumulation curve of an anastomosis.

The anonymous expert survey was then conducted with *n* = 66 participants from multiple centers. This included 34 surgeons, all working in general or visceral surgery, 8 (24%) were female and 26 (76%) were male. They had been working in their professional field since the beginning of residency for a mean of 11.9 ± 7.0 years. Furthermore, 28 scientists participated, 23 of them computer scientists (82%), 7 (25%) of them were female and 21 (75%) male. They had been working as scientists since graduation for a mean of 7.6 ± 7.8 years. Participants with “other” professional backgrounds were excluded from analysis (*n* = 4).

Clinical relevance for morbidity and mortality as rated by surgeons was highest for the feature category “surgical skill and quality of performance” (9.0 ± 1.3) with an average rating of 6.8 ± 1.1 over all categories and also for long-term outcome (8.2 ± 1.8) with an average rating of 5.8 ± 1.2 over all categories. Technical feasibility of automatic recognition with ML as rated by scientists was highest for the “Instrument” category (8.5 ± 1.7) with an average feasibility rating over all categories of 6.8 ± 1.3. Figure 3 gives an overview of the ratings for all categories.

Surgeons and scientists gave the highest ranking of overall relevance within their respective category to the same surgomic features “intraoperative adverse events” (surgical field category), “action performed with instrument” (instrument category), “vital sign monitoring data” (anesthesiological category), “difficulty of surgery” (surgical skill category), “familiar surgeries performed / experience with surgery” (surgical team category), and “emotional atmosphere in OR” (environment category). Table 2 gives an overview of the ranking for all surgomic features.

**Table 2:** Overall priority of surgomic features

Category	Surgical field		Instrument		Temporal course of procedure		Patient	
Ranking	Surg	Sci	Surg	Sci	Surg	Sci	Surg	Sci
1st	Intraoperative adverse events (2.4 ± 2.0)	Intraoperative adverse events (3.5 ± 2.7)	Action performed with instrument (1.8 ± 0.9)	Action performed with instrument (1.9 ± 1.2)	Duration of surgery (1.5 ± 0.6)	Order of performed phases/steps (1.6 ± 0.7)	Texture of liver (3.2 ± 1.8)	Frozen section result (2.9 ± 1.9)
2nd	Bloodiness (3.6 ± 2.3)	Presence of anatomic structure (3.6 ± 2.5)	Instrument usage (3.5 ± 1.4)	Presence of instrument (2.9 ± 1.8)	Order of performed phases/steps (1.8 ± 0.8)	Duration of surgery (1.8 ± 0.8)	Texture of pancreatic gland (3.3 ± 1.5)	Texture of liver (3.1 ± 1.8)
3rd	Action performed with anatomic structure (3.8 ± 1.9)	Action performed with anatomic structure (3.8 ± 1.9)	Location of instrument (3.7 ± 1.7)	Instrument usage (3.3 ± 1.4)	Duration of inside/outside sequences (2.8 ± 0.5)	Duration of inside/outside sequences (2.6 ± 0.6)	Texture of lung (3.4 ± 2.3)	Texture of pancreatic gland (3.1 ± 1.6)
4th	Presence of anatomic structure (5.2 ± 2.7)	Location of anatomic structure (4.0 ± 2.5)	Presence of instrument (3.7 ± 2.3)	Location of instrument (3.4 ± 1.2)	n/a	n/a	Frozen section result (4.0 ± 2.0)	Pancreatic duct diameter (3.4 ± 1.5)
5th	Location of anatomic structure (5.6 ± 2.4)	Bloodiness (4.2 ± 1.9)	Instrument pressure or flow (4.4 ± 2.1)	Instrument pressure or flow (5.4 ± 1.5)	n/a	n/a	Gastric tube diameter (4.5 ± 1.8)	Volume of remnant gland (4.5 ± 2.1)
6th	Hyperspectral imaging of anatomic structures (5.8 ± 2.3)	Colors of anatomic structures (6.0 ± 2.1)	Instrument open/closed (5.3 ± 1.3)	Instrument open/closed (5.6 ± 1.3)	n/a	n/a	Pancreatic duct diameter (4.6 ± 1.8)	Texture of lung (5.3 ± 1.5)
7th	Indocyanine green accumulation curve of perfusion in anatomic structures (5.9 ± 2.4)	Hyperspectral imaging of anatomic structures (6.4 ± 2.3)	Number of camera cleanings (5.6 ± 1.2)	Number of camera cleanings (5.6 ± 1.7)	n/a	n/a	Volume of remnant gland (4.9 ± 2.2)	Gastric tube diameter (5.7 ± 1.6)
8th	Smokiness (6.3 ± 2.2)	Indocyanine green accumulation curve of perfusion in anatomic structures (6.5 ± 1.9)	n/a	n/a	n/a	n/a	n/a	n/a
9th	Colors of anatomic structures (6.5 ± 2.0)	Smokiness (7.0 ± 2.0)	n/a	n/a	n/a	n/a	n/a	n/a
10th	n/a	n/a	n/a	n/a	n/a	n/a	n/a	n/a
11th	n/a	n/a	n/a	n/a	n/a	n/a	n/a	n/a

Category	Anesthesiological data		Surgical skill and quality of performance		Surgical team		Environment	
Ranking	Surg	Sci	Surg	Sci	Surg	Sci	Surg	Sci
1st	Vital sign monitoring data (1.1 ± 0.3)	Vital sign monitoring data (1.3 ± 0.6)	Difficulty of surgery (3.3 ± 3.1)	Difficulty of surgery (3.5 ± 3.1)	Familiar surgeries performed / Experience with surgery (2.3 ± 2.0)	Familiar surgeries performed / Experience with surgery (2.7 ± 2.0)	Emotional atmosphere in OR (1.6 ± 0.8)	Emotional atmosphere in OR (1.8 ± 0.8)
2nd	Blood gas analysis (2.2 ± 0.6)	Blood gas analysis (2.1 ± 0.7)	Suture quality of anastomosis (4.6 ± 3.0)	Tissue handling (4.5 ± 2.2)	Quality of team communication (3.3 ± 1.9)	Quality of team communication (3.1 ± 2.2)	Noises in the OR (1.8 ± 0.7)	Noises in the OR (1.8 ± 0.7)
3rd	Drug administration (2.7 ± 0.5)	Drug administration (2.6 ± 0.5)	Tissue handling (5.4 ± 2.9)	Suture quality of anastomosis (4.6 ± 2.4)	Surgeries performed as a team (3.5 ± 2.4)	Surgeries performed as a team (3.8 ± 2.1)	Temperature in OR (2.6 ± 0.7)	Temperature in OR (2.4 ± 0.8)
4th	n/a	n/a	Tension of anastomosis (5.5 ± 2.9)	Efficiency (5.6 ± 2.7)	Stress level (4.4 ± 1.9)	Fastness of decision-making (4.5 ± 2.1)	n/a	n/a
5th	n/a	n/a	Efficiency (6.2 ± 3.4)	Tissue exposure (5.6 ± 2.9)	Fastness of decision-making (4.8 ± 2.0)	Stress level (4.6 ± 2.0)	n/a	n/a
6th	n/a	n/a	Extend of dissection/resection (6.4 ± 2.8)	Extend of dissection/resection (5.7 ± 3.4)	Number of team members (6.3 ± 2.4)	Position of team members (6.0 ± 2.5)	n/a	n/a
7th	n/a	n/a	Tissue exposure (6.5 ± 2.7)	Tension of anastomosis (5.8 ± 3.0)	Heart rate (6.5 ± 2.0)	Number of team members (6.1 ± 2.4)	n/a	n/a
8th	n/a	n/a	Depth perception (6.6 ± 2.7)	Bimanual dexterity (6.4 ± 2.9)	Position of team members (6.9 ± 1.8)	Heart rate (6.8 ± 2.0)	n/a	n/a
9th	n/a	n/a	Autonomy (6.9 ± 3.8)	Depth perception (7.4 ± 3.1)	Voice frequency (7.0 ± 1.8)	Voice frequency (7.3 ± 1.5)	n/a	n/a
10th	n/a	n/a	Bimanual dexterity (7.2 ± 2.8)	Autonomy (8.3 ± 2.6)	n/a	n/a	n/a	n/a
11th	n/a	n/a	Force of stomach pull-up (7.4 ± 2.7)	Force of stomach pull-up (8.8 ± 2.2)	n/a	n/a	n/a	n/a

Features are sorted per category as ranked by surgeons (Surg) and scientists (Sci)

In the free text answers for new surgomic features a total of *n* = 34 new features were mentioned. Most ideas for new features were given in the “anesthesiological data” category and the “surgical field” category. Here, eight new feature ideas were added each, e.g. “degree of relaxation/depth of narcosis” and “anastomosis technique (in general which techniques were used)”, respectively. In the “anesthesiological data” category “ventilation parameters” were mentioned by *n* = 3 participants. The “instrument” category followed with five new feature ideas, e.g. “movement of instrument (trajectory)” (*n* = 2 participants). In this category one participant also suggested an intraoperative assistance based on the surgomic features, i.e. the “visual highlight of potentially dangerous instruments when inserted”. In category “temporal course of procedure” *n* = 6 participants suggested the duration of certain phases as a new feature, which was originally listed by us as part of the feature “duration of surgery”, but should be a separate feature. The full list of the free text answers for new surgomic feature ideas can be found in supplement 1.

## Discussion

### Conceptualizing Surgomics

With Surgomics we coined a neologism to comprehensively describe a surgical procedure based on automatic analysis of intraoperative data. This is in analogy to Radiomics, where the mere idea that “images are more than pictures, they are data” is far from new [61]. The specific challenge of surgical data is taking temporal data and the whole surgical team into account. Here, the representation of temporal data is far from trivial and actually an open research question. Data has been systematically collectedin the operating room to improve patient care [24], ML has been used for surgical skill assessment [62]. However, with Surgomics our aim is to go beyond assessment of an individual surgeon’s skill. The aim is to approach the massive amount of data that is generated daily in the operating rooms with a holistic perspective on all possible sources of data and provide a conceptual framework for future developments of automatic analysis of this data keeping the improvement of surgical patient care in focus. Thereby, Surgomics can also add a fine granular, objective analysis to previous approaches that proved the importance of intraoperative adverse event classification [44, 45].

We thus gathered a group of 19 specialists from both surgery and (computer) science to flesh out the concept of Surgomics. As a result we came up with a first collection of 52 surgomic features that may serve as surrogate parameters for the quality of patient care and defined eight categories to group them (Table 1).

When validating this conception with a broader audience of surgeons and (computer) scientists we found the rating of relevance for morbidity and mortality for the category “surgical skill and quality of performance” as the only one with a significant difference between surgeons and scientists (*p* = 0.002). In contrast to surgeons, scientists rated the category “anesthesiological data” as most relevant for morbidity and mortality and not “surgical skill and quality of performance”. We assume this surprising agreement to result from a selection bias, because the participating (computer) scientists are members of surgical-technical societies and/or have a long track record of scientific work in computer-assisted surgery. This highlights that a close scientific exchange between clinicians and computer scientists is beneficial or even mandatory to ensure optimal planning of the next steps to differentiate and develop individual surgomic features.

Surgeons rated the category “surgical skill and quality of performance” highest in relevance for morbidity and mortality as well as for long-term (oncological) outcome. The feasibility of this category, however, was rated penultimate by scientists. Interestingly, the surgomic feature rated with highest overall priority in this category was “difficulty of surgery” far beyond sub-dimensions of the established GOALS-score such as “tissue handling”, “tissue exposure” and “efficiency”.

A better relation between clinical relevance and technical feasibility was found for “anesthesiological data” with high ratings for morbidity and mortality, but not for long-term (oncological) outcome. At the same time this category had the second highest rating for feasibility, rendering it a good candidate for further investigation. When rating the features surgeons and scientists agreed on “vital sign monitoring” having the highest relevance. Here it would be interesting to investigate which parameter(s), such as heart rate, arterial blood pressure, central venous pressure or ventilation parameters are of highest relevance.

Another promising category is “patient” with the second highest rating in long-term (oncological) outcome and third in morbidity and mortality. Whereas the technical feasibility was rated lower, surgomic features that could be easily obtained with computer vision from laparoscopic video such as “texture of liver”, “texture of lungs” and “texture of pancreatic gland” had high relevance ratings.

Other feature categories that have been of high interest in the scientific literature on computer vision for laparoscopic surgery, namely “surgical field”, “instrument” and “temporal course of procedure” received only medium ratings for clinical relevance, but comparatively high technical feasibility. Because of that high technical feasibility these categories should also be a matter of future investigations, in order to create a success story of the Surgomics concept that would boost motivation to tackle more challenging features for example from the category of “surgical skill and quality of performance”. Good feature candidates would be the highly rated “intraoperative adverse events”, “bloodiness”, “action performed with anatomic structure”, “action performed with instrument”, and “instrument usage”.

The category “environment” was neither rated high in clinical relevance nor in technical feasibility and will thus have a low priority for future investigations.

Although many surgomic features have been identified and described, our list is far from complete. The free text questions for more surgomic feature ideas already revealed 34 new features. Furthermore, a relevant proportion of our 52 surgomic features is to be extracted from surgical video in minimally invasive surgery. More surgomic features from various sources of raw data will be identified in surgical and scientific discussions as well as during the development process of other features. Either way, the suitability of every surgomic feature for a certain application has to be validated thorougly before introduction into clinical practice.

A limitation of our study is that in our group only general and visceral surgeons participated, potentially missing features that would be important for other specialties such as cardiac or thoracic surgery. Also, other parts of the OR team such as anesthesiologists and scrub nurses have not been involved in our study, but probably will come up with other surgomic features in future discussions.

Furthermore, some definitions of features and their categories can be hard to distinguish. The instrument category could be perceived as part of the surgical field category, but we chose to define a different category to distinguish what happens in the surgical field and which instruments perform these activities integrating data that is not derived from the video, but from connected medical devices (e.g. electrocautery).Other surgomic features like the “emotional atmosphere in the OR”, which was assigned to the “environment” category, could as well be assigned to the “surgical team” category.

### Pre-, intra- and postoperative use

Based on the idea of using intraoperative data for improvement of patient care, numerous potential applications for Surgomics along the whole treatment path arise. Preoperative information about the patient (comorbidities, previous surgeries) as well as their disease (tumor stage, lymph node involvement) is routinely collected. It is obvious that this information will influence the intraoperative course. For example, previous surgeries may result in more or less severe abdominal adhesions and some surgical centers may limit minimally invasive oncologic surgery to T2/T3 cancers. Antithrombotic medication may turn a straightforward procedure into a more demanding one. Whereas established metrics such as overall procedure time or intraoperative blood loss can hint towards those correlations, surgomic features such as bloodiness or length of certain key steps of a procedure can give deeper insights into correlations or even causality.

Intraoperatively, assistance-systems based on Surgomics may support learning surgeons with real time analysis and a recommendation of what other surgeons would do in the same situation. Furthermore, Surgomics may help with intraoperative decision-making for individual patients by correlating quantitative measurements such as gastric tube width or indocyanine green (ICG) perfusion quantification with other procedure or postoperative outcome parameters.

Postoperatively, the aim of Surgomics is to predict life-threatening complications. Even if the blood loss as estimated by the anesthesiologist is low, the “bloodiness” of an operation may be high in a specific part of the procedure leading to postoperative hematoma and infection. Even if the subjective impression of ICG perfusion of an anastomosis seems good, the accumulation of the perfusion in combination with an ruptured suture may result in anastomotic insufficiency. By applying the concept of Surgomics, clinical researchers may find correlations that can be used to develop clinical decision support systems based on those surgomic features. Here, the aim would be to predict complications of the individual patient before they become clinically apparent and to warn surgeons accordingly [56] to reduce the rate of “failure to rescue” [63].

On an individual surgeon level, Surgomics may be used as a foundation for an objective quantitative metric of surgeon performance. By incorporating not only audiovisual data from the OR but other sensors from medical devices, vital sign monitoring, etc. Surgomics is a more comprehensive concept than surgical sabermetrics. Here, Surgomics may be used for regular assessment of surgical residents to objectify their progress and tailor their training accordingly. For experienced surgeons, Surgomics may help to establish data-driven morbidity and mortality analysis and bring up hypotheses for performance improvement by providing deeper insights into correlation of intraoperative activities with postoperative outcomes. Also, in contrast to global scores of overall performance, Surgomics again may help surgeons by breaking down potential reasons for a case being exceptionally difficult by giving insights into factors of patient, surgeon, team, and environment. In this manner, Surgomics can be a methodological approach to use ML for automatic surgical quality assessment, or to better classify and compare procedures in a learning curve.

Furthermore, Surgomics may become a tool to automate the comprehensive description and classification of a surgical procedure, similar to TNM in pathology, going beyond already promising approaches to categorize intraoperative adverse events [44, 45]. Surgomics thus may serve to improve comparability of clinical performance and procedures in surgical trials.

### Ethical considerations

When introducing a concept that provides comprehensive insights into the intraoperative course and might even produce objective data of inadequate surgeon performance, difficult ethical questions arise. Here, we are having the same situation as e.g. in aviation with flight recorders (black box) or with CIRS (critical incident reporting systems) in hospitals. If in analogy further approaches regarding the assessment of the surgeon’s skill and the quality of performance are to be made, ethical considerations are extremely important. There is no doubt that performance data needs to be collected objectively [14] with the surgical team members being fully aware of the recording, the further use of the data and the possible consequences of inadequate surgical performance. Moreover, concerns regarding privacy and litigation need to be addressed as investigated in a study on perception of the OR blackbox® [64]. However, nowadays some authors consider a routine video recording of surgeries an ethical duty and a standard of care with potential benefits for e.g. safety, fairness and candor [65]. Already in 2011, the American Board of Medical Specialties (ABMS) defined requirements that a systematic tracking of the surgeon’s performance is necessary [12]. Furthermore, in case there are legal implications, it might even be helpful to have proper documentation of the performed activities to protect surgeons. Apart from the legal aspects, video-based coaching sessions with senior surgeons focusing on the operative technique have already been successfully introduced [66]. Using Surgomics for an automated assessment of surgical skills with anonymous, constructive feedback based on expert knowledge may even be less intimidating than personal feedback from a surgical supervisor a surgeon is dependent on. Nevertheless, we are convinced that objective data of performance can open the room for discussion and improvement. This way, a positive culture regarding mistakes is to be established with surgeons being aware of their own performance level, strengths and room for improvement.

### Next steps

The next step to realize the concept of Surgomics would be the automatic recognition of features. Here, our work not only introduces a concept, but provides a conceptual framework for future research that can help to shape the process of intraoperative data analysis and provides guidance for surgeons and computer scientists alike. Surgeons with ideas for new surgomic features find the technical feasibility of feature categories rated by (computer) scientists and (computer) scientists that want to apply their technology to surgery find the clinical relevance of surgomic feature categories rated by surgeons. Based on the analysis of our survey with experienced professionals from surgery and (computer) science, surgomic features like “vital sign monitoring” (“anesthesiological data” category) as well as texture of liver, lung, and pancreas (“patient” category) have a suitable combination of expected clinical relevance and technical feasibility. Furthermore, the surgomic features “intraoperative adverse events”, “bloodiness”, “action performed with anatomy” (“surgical field” category), “action performed with instrument”, and “instrument usage” (“instrument” category), should be investigated. Because of their high technical feasibility and the already active scientific community in the field of laparoscopic computer vision, they represent “low hanging fruits” to create success stories for Surgomics.

This success also depends on open source availability of annotated surgical data and ML models. Annotated surgical data can be used in international ML challenges to validate algorithms for automatic extraction of surgomic features from surgical data. The openly available ML models could then be used by surgical researchers at different centers to investigate the use of surgomic features as new parameters in clinical research. This in turn would stimulate discussions within surgical societies to investigate more surgomic features and their application to clinical problems similar to current developments in Radiomics and Genomics for precision medicine.

## Conclusion

The aim of Surgomics is to predict a patient's morbidity, mortality and long-term outcome using ML to derive personalized information from comprehensive intraoperative sensor data and combining it with pre- and postoperative information. With a multidisciplinary group of experts, we defined this concept, and came up with 52 features in eight categories that were rated by experienced professionals from surgery and (computer) science. This is a first step to establish Surgomics as the surgical answer to Radiomics and Genomics for improving surgical patient care.

## Supplementary Information

Below is the link to the electronic supplementary material.

Supplementary file1 (PDF 114 kb)
