# Supplementary material for: Surgomics: personalized prediction of morbidity, mortality and long-term outcome in surgery using machine learning on multimodal data
Source: Surg Endosc. 2022 Sep 28;36(11):8568–91. doi: 10.1007/s00464-022-09611-1 (PMC9613751; doi:10.1007/s00464-022-09611-1)
Supplement: Supplementary file 1 — Supplementary file1 (PDF 114 kb) [file 464_2022_9611_MOESM1_ESM.pdf]

Supplement 1 - Free text answers in expert questionnaire

In our expert questionnaire participants were asked to suggest additional surgomic features for each surgomic feature category within a free text field. The original answers are listed below.

New surgomic features

1. Surgical field
    - Adhesions
    - Presence of foreign bodies (e.g. compress, needle)
    - Anastomosis technique (in general which techniques were used)
    - Color of esophageal mucosa
    - Dissection with respect to anatomic layers/structures and their reconstruction
    - Number of previous operations
    - Known anatomic variants (e.g., right hepatic artery from superior mesenteric artery)
    - Status post removal of organs/structures
2. Instrument
    - Material usage such of clips, gauze or needles
    - Movement of instrument (trajectory) (n=2)
    - Instrument type
    - Experience of team members with specific types of instruments
    - Type of instrument in connection with surgical phase, enabling recognition of e.g. an error made by the surgical team

3. Temporal course of procedure
    - Duration of steps/phases (n=6, already included in detailed description of our surgomic feature nr. 17 “duration of surgery”)
    - Date and starting time of surgery, e.g. Mondays or in the evening the procedure might be less accurate
    - Deterioration from standard procedure
4. Patient
    - Texture/color of intestine
    - Texture of stomach
5. Anesthesiological data
    - Amount / type of blood products given to patients during surgery (n=3), e.g. erythrocytes, platelets, PPSB
    - Amount of catecholamines (especially at end of surgery) (n=2)
    - Ventilation parameters (n=3)
    - Difficulty of ventilation (n=2)
    - History of the patient
    - Data of anesthesiological devices, e.g. central venous line, peridural catheter
    - Degree of relaxation / depth of narcosis
    - Lab test results (pre- and intraop)
6. Surgical skill and quality of performance
    - Number of surgeries performed of this type
7. Surgical team
    - Non-verbal communication aspects
    - Modes of interaction between team members
    - Quality of decision making

8. Environment
    - Room condition (e.g. old or modern)
    - Number of times someone is entering the OR
    - Disturbances
    - Number of phone calls interrupting the surgeon

Suggestions for intraoperative assistance based on surgomic features

- Instrument
    - Visual highlight of potentially dangerous instruments (e.g., scissors) when inserted
    - Highlight of potential harmful application of instrument, e.g., cautery while bipolar forceps manipulates bowel
